# IntelliCare: An Eclectic, Skills-Based App Suite for the Treatment of Depression and Anxiety

**DOI:** 10.2196/jmir.6645

**Published:** 2017-01-05

**Authors:** David C Mohr, Kathryn Noth Tomasino, Emily G Lattie, Hannah L Palac, Mary J Kwasny, Kenneth Weingardt, Chris J Karr, Susan M Kaiser, Rebecca C Rossom, Leland R Bardsley, Lauren Caccamo, Colleen Stiles-Shields, Stephen M Schueller

**Affiliations:** ^1^ Center for Behavioral Intervention Technologies (CBITs) Department of Preventive Medicine Northwestern University Chicago, IL United States; ^2^ Department of Preventive Medicine Northwestern University Chicago, IL United States; ^3^ Audacious Software Chicago, IL United States; ^4^ HealthPartners Institute Minneapolis, MN United States

**Keywords:** mHealth, eHealth, mobile health, depression, anxiety

## Abstract

**Background:**

Digital mental health tools have tended to use psychoeducational strategies based on treatment orientations developed and validated outside of digital health. These features do not map well to the brief but frequent ways that people use mobile phones and mobile phone apps today. To address these challenges, we developed a suite of apps for depression and anxiety called IntelliCare, each developed with a focused goal and interactional style. IntelliCare apps prioritize interactive skills training over education and are designed for frequent but short interactions.

**Objective:**

The overall objective of this study was to pilot a coach-assisted version of IntelliCare and evaluate its use and efficacy at reducing symptoms of depression and anxiety.

**Methods:**

Participants, recruited through a health care system, Web-based and community advertising, and clinical research registries, were included in this single-arm trial if they had elevated symptoms of depression or anxiety. Participants had access to the 14 IntelliCare apps from Google Play and received 8 weeks of coaching on the use of IntelliCare. Coaching included an initial phone call plus 2 or more texts per week over the 8 weeks, with some participants receiving an additional brief phone call. Primary outcomes included the Patient Health Questionnaire-9 (PHQ-9) for depression and the Generalized Anxiety Disorder-7 (GAD-7) for anxiety. Participants were compensated up to US $90 for completing all assessments; compensation was not for app use or treatment engagement.

**Results:**

Of the 99 participants who initiated treatment, 90.1% (90/99) completed 8 weeks. Participants showed substantial reductions in the PHQ-9 and GAD-7 (*P*<.001). Participants used the apps an average of 195.4 (SD 141) times over the 8 weeks. The average length of use was 1.1 (SD 2.1) minutes, and 95% of participants downloaded 5 or more of the IntelliCare apps.

**Conclusions:**

This study supports the IntelliCare framework of providing a suite of skills-focused apps that can be used frequently and briefly to reduce symptoms of depression and anxiety. The IntelliCare system is elemental, allowing individual apps to be used or not used based on their effectiveness and utility, and it is eclectic, viewing treatment strategies as elements that can be applied as needed rather than adhering to a singular, overarching, theoretical model.

**Trial Registration:**

Clinicaltrials.gov NCT02176226; http://clinicaltrials.gov/ct2/show/NCT02176226 (Archived by WebCite at http://www.webcitation/6mQZuBGk1)

## Introduction

Psychological treatments, although effective in treating depression and anxiety [1,2] and preferred to psychopharmacological treatments by two-thirds of patients in primary care [3-6], are inaccessible by up to 75% of people due a variety of barriers including lack of availability of services, time constraints, transportation problems, and cost [7,8]. Furthermore, due to the high prevalence of both depression and anxiety disorders, we will likely never be able to meet the demand for services with standard one-on-one intensive treatments [9].

To overcome these barriers and meet treatment needs, a wide variety of Web-based treatments have been developed and shown to be highly effective in the treatment of depression and anxiety, particularly when coupled with some human support to promote adherence and enhance outcomes [10,11]. These programs, leveraging the strengths of computer-accessed Web programs in providing information, have strong psychoeducational components along with some interactional components that function much like worksheets [12].

Substantially less is known about the use of mobile phone apps for the treatment of anxiety and depression [13]. The use of mobile phones is rapidly increasing around the world, with 72% of Americans using mobile phones in early 2016—up from just 35% in early 2011 [14]. People use their phones for a variety of functions including supporting their health. A recent US national survey indicated that more than half of mobile phone users (58%) have downloaded at least one health-related mobile app [15], and more than half of outpatient psychiatry patients report wanting to use mental health apps [16].

A key challenge is designing apps to be useful and usable. The use of mobile phones and mobile phone apps tends to differ considerably from the use of desktop computers and websites with considerations ranging from screen real estate to where and when they are used. Although websites can be designed to be responsive and accessible via multiple devices, including mobile phones, if interactional styles remain highly didactic this approach fails to consider the unique affordances and challenges of using mobile phone apps [17]. For example, apps that require lengthy engagement times, or that have deeper navigation to provide multiple features do not fit well with how people use mobile apps. Typically, popular apps serve singular purposes, such as searching for restaurants or businesses, managing flights, or posting pictures. People tend to use apps in very short bursts of time, sometimes frequently [18,19]. Thus, apps tend to use simple interactions, are quick to use, and support a single or limited set of related tasks.

This creates a number of problems for the design of interactive apps aimed at improving mental health. Most apps provide psychoeducation via text or video, or simply facilitate logging symptoms or mood, requiring the bulk of treatment to be conducted elsewhere, such as face-to-face or through computer interfaces [20,21]. Given that for many people mobile phones represent their only access to the Internet [14], mobile phone intervention apps that do not require other devices will be important for broader dissemination. Furthermore, solutions that are independent of other treatment sources and use the app and mobile phone as the platform to enable new digitally enabled services can help provide a more distinct option from traditional approaches to mental health service delivery. Indeed, psychoeducational or curricular approaches tend to lean heavily on content and concepts from psychotherapy developed for face-to-face therapy, such as cognitive-behavioral therapy [22], which results in a skeuomorph, tunneling the vision of developers to the exclusion of innovation and limiting the options for consumers [23]. To address these challenges, we designed IntelliCare using the Behavioral Intervention Technology (BIT) Model as a framework [24] to be elemental, skills-based, and eclectic, and more consistent with a frequent, dynamic style of real-world interactions rather than a weekly, didactic model drawn from face-to-face practices.

IntelliCare is *elemental* in that it is a suite of apps, each supporting a single skill or using a single interactional style to support acquisition of a set of skills with a focused theoretical aim related to depression or anxiety (eg, goal setting, cognitive restructuring, exposure). This is consistent with the US Institute of Medicine’s report outlining a framework for establishing evidence-based standards for psychosocial interventions, which recommended identifying effective treatment elements [25]. IntelliCare is *skills-based,* emphasizing in-the-moment practice of new skills over psychoeducational or curricular approaches. Most IntelliCare apps prioritize “doing” over knowledge acquisition. Thus, the user is prompted to *do* the task right at the beginning of the interaction. For most apps, there is no explanation prior to engagement. Apps were designed to make the interaction as intuitive as possible. Minimal didactic content relegated to a “Help” section in almost all apps. Apps are designed for brief, frequent interactions to be used in-the-moment, consistent with prevailing mobile phone use patterns using common interaction elements (eg, logging, checklists, reminders, simple gamification). IntelliCare is *eclectic,* as current evidence-based behavioral and psychological strategies come from diverse theoretical frameworks (eg, acceptance and commitment therapy, cognitive-behavioral therapy, positive psychology, problem-solving therapy). This is consistent with a growing movement arguing that treatment elements should be applied based on patient variables and preferences, rather than the theoretical orientation from which they evolved [26,27]. Therefore, the IntelliCare app suite is intended to be a framework that is extensible and modifiable.

Most of these apps have been available on the Google Play store since September 2014. A recent paper describing the first 10,131 downloads showed good use, with the mean app launch rate ranging from 3.1 to 17.0, and mean time from first to last launch ranging from 13.0 to 25.3 days, depending on the app [28]. However, evaluation of clinical benefit from that sample was lacking. Since the initial Google Play deployment, another app, BoostMe, was added after extensive user research and usability testing, which focused on behavioral activation [29].

In addition to evaluating clinical efficacy, the other major departure between the Google Play store deployment and this study is the inclusion of coach support. The addition of a coach to support treatment and adherence with the program is important, given that coaching appears to improve outcomes with mobile interventions [30]. Although it is true that many stand-alone apps exist in app marketplaces, many offerings are beginning to include human support (eg, Coach.me, Ginger.io, Joyable, Lantern). Many human support models have been developed in the context of more psychoeducational programs, for example, [31,32]. Our objective was to develop and evaluate a lean, low-effort coaching protocol aimed at efficiently promoting use and positive outcomes that would be consistent with the interactional style of these apps.

The primary aim of this single-arm pilot study was to evaluate the change in depression and anxiety, as well as app use, during 8 weeks of IntelliCare supported by low-intensity coaching.

## Methods

### Participants

Participants were recruited from March 2015 to March 2016 from a variety of sources including the Minnesota-based health care system, HealthPartners, online (eg, ResearchMatch, Craigslist, Reddit, clinicaltrials.gov, social media advertising) and community (eg, print advertisements posted on public transportation, media) advertising, and clinical research registries. Recruitment materials informed participants that the study was examining the use of mobile phone apps to teach self-management skills for depression and anxiety.

Participants were included in this single-arm field trial if they exhibited depressive symptoms indicated by a score of 10 or higher on the Patient Health Questionnaire-9 (PHQ-9) [33], or anxiety symptoms indicated by a score of 8 or higher on the Generalized Anxiety Disorder-7 (GAD-7) questionnaire [34]; were 18 years of age or older (age 19 if in Nebraska, given age of consent); could speak and read English, living in the United States; and owned and were familiar with an Internet-ready Android mobile phone with data and text plans. The PHQ-9 and GAD-7 closely match the Diagnostic and Statistical Manual of Mental Disorders (DSM-5) criteria for major depressive disorder and generalized anxiety disorder, respectively. Furthermore, these measures are widely used in primary care settings and useful for identifying and monitoring depression and anxiety in clinical and general populations [35-37]. Thus, these inclusion criteria are similar to what might be expected to identify people at need for real-world deployments of similar treatment options. Participants were excluded if they had any visual, hearing, voice, or motor impairments that would prevent completion of study procedures; met diagnostic criteria for a severe psychiatric disorder (eg, bipolar disorder, psychotic disorder, dissociative disorder) or any other diagnosis for which this trial was either inappropriate or dangerous; exhibited severe suicidality including having ideation, a plan, and intent; had initiated or changed antidepressant or antianxiolytic pharmacotherapy in the previous 14 days; or had used any of the IntelliCare apps for more than 1 week in the last 3 months.

### Procedures

The IntelliCare field trial was approved by the Northwestern University institutional review board and monitored by an independent data and safety monitoring reviewing board. People interested in participating completed an initial Web-based or telephone screener and, if eligible to continue, were sent the consent form. Once signed, the consent form was reviewed over the telephone with research staff to ensure understanding, after which people received an eligibility assessment consisting of a phone interview and Web-based questionnaires. People meeting eligibility criteria were offered participation in the field trial. As part of the field trial, participants received 8 weeks of coaching aimed at helping them use the IntelliCare app suite, and received additional assessments at weeks 4 and 8. Participants were compensated for completing all assessments and could earn up to US $90. Payment was not tied to app use or engagement in coaching.

### IntelliCare Apps

The IntelliCare program consisted of 14 apps in total, including 13 clinical apps designed to improve symptoms of depression and anxiety through efficacious treatment strategies, and the “Hub” app, which coordinates a user’s experience with the clinical apps [28]. A description of each clinical app can be found in Table 1.

**Table 1:** Description of IntelliCare apps.

App	Behavioral strategy	Description
IntelliCare Hub		Manages messages and notifications from the other apps within the IntelliCare collection.
Aspire	Personal values and goal setting	Guides user to identify the values that guide one’s life and the actions (or “paths”) that one does to live that value. Helps keep track of those actions throughout the day and supports the user in living a more purpose-driven and satisfying life.
Day to Day	Psychoeducation and prompts	Delivers a daily stream of tips, tricks, and other information throughout the day to boost the user’s mood. Prompts the user to work on a particular theme each day and every week; learn more about how to effectively cultivate gratitude, activate pleasure, increase connectedness, solve problems, and challenge one’s thinking.
Daily Feats	Goal setting	Encourages the user to incorporate worthwhile and productive activities into the day. Users add accomplishments to the Feats calendar, where they can track their positive activity streaks and level up by completing more tasks. Helps motivate users to spend their days in more meaningful, rewarding ways to increase overall satisfaction in life.
Worry Knot	Emotional regulation and exposure	Teaches the user to manage worry with lessons, distractions, and a worry management tool. Provides a guided tool to address specific problems that a user cannot stop thinking about and provides written text about how to cope with “tangled thinking.” Presents statistics about progress as the user practices coping with worry, gives daily tips and tricks about managing worry, and provides customizable suggestions for ways to distract oneself.
ME Locate	Behavioral activation	Provides a personal map for finding and saving user’s mood-boosting locations. Assists the user in finding and remembering these places to help them make plans, maintain a positive mood, and stay on top of responsibilities.
Social Force	Social support	Prompts the user to identify supportive people in their lives, and provides encouragement for the user to get back in touch with those positive people.
My Mantra	Self-affirmations and positive reminiscence	Prompts the user to create mantras (or repeatable phrases that highlight personal photo strengths and values and can motivate one to do and feel good) and construct virtual albums to serve as encouragement and reminders of these mantras.
Thought Challenger	Cognitive reframing	Guides the user through an interactive cognitive restructuring tool to examine thoughts that might exaggerate negative experiences, lead one to be overcritical, and bring down one’s mood. Teaches the user to get into the habit of changing perspective and moving toward a more balanced outlook on life.
iCope	Proactive coping	Allows the user to send oneself inspirational messages and reassuring statements, written in their own words, to help the user get through tough spots or challenging situations.
Purple Chill	Relaxation	Provides users with a library of audio recordings to relax and unwind. Teaches a variety of relaxation and mindfulness practices to destress and worry less.
MoveMe	Exercise for mood	Helps the user select exercises to improve mood. Provides access to curated exercise videos and to written lessons about staying motivated to exercise. Allows the user to schedule motivational exercise time for oneself throughout the week.
Slumber Time	Sleep hygiene	Prompts the user to complete sleep diaries to track sleep. Provides a bedtime checklist intended to clear one’s mind before going to sleep. Provides audio recordings to facilitate rest and relaxation. Features an alarm clock function.
Boost Me	Behavioral activation	Encourages users to select and schedule positive activities (“boosts”) when they notice a drop in mood and to track positive activities they note positively impacting their mood. Includes animated mood tracking for pre or post positive activities, calendar integration, and suggested activities that are auto-populated based on past mood improvement.

The apps prioritize the use of an interactive tool to help the user learn skills and engage in the treatment strategy. These tools are typically either on the first screen or accessible directly from it, thereby requiring little navigation. Tools are designed to be intuitive, requiring few instructions, and each app contains brief “tips” on the home screen to guide users through their first interaction with a new tool. Any didactic or psychoeducational material is usually available under the “Help” menu, usually in a template form that includes the following topics: (1) Why use this app, (2) How can this app help me, (3) How to use this app, (4) How often to use this app, (5) What might get in the way of using this app, and (6) Call to Action or What to do now. The clinical apps are available for free download on the Google Play Store.

The Hub app provides a number of organizing functions for users. It consolidates notifications, so those who have multiple IntelliCare apps have a single notification view, and all IntelliCare apps are listed and can be downloaded through the Hub app. The Hub app also provides recommendations for new apps (2 per week). In this trial, weekly recommendations for new apps were made randomly to support the development of the recommendation engine. Once the planned recommender engine is completed, app recommendations will be derived from algorithms of patterns of use data to identify apps that the person will most likely use and find useful. Participants were also free to explore and download apps on their own. Participants were not restricted in the number of apps they could have on their phone or use at any given point in time. However, for this trial, participants were guided to choose 1 or 2 apps to focus on per week to provide them with an opportunity to build proficiency in these 1 or 2 skills. To support this model, the Hub app also contains a feature that allows users to identify 1 or 2 of the IntelliCare apps as “primary,” meaning they are the main treatment focus for a given week, with the goal of having them try a variety of apps during the study period and learn several skills that help them accomplish their goals. Apps identified as primary are highlighted on the app’s home screen.

### Coaching Protocol

The coaching protocol was based on aspects of the Efficiency Model of BIT Support [38] and supportive accountability [39]. Coaching was aimed primarily at encouraging participants to try the apps recommended to them through the Hub app. Coaches also answered questions about how to use the tools found in the apps and the rationale behind the skills taught by the apps, encouraged application of skills in daily life, and provided some technical support as needed. Coaching began with an initial 30- to 45-minute engagement phone call to establish goals for mood and anxiety management, ensure the participant could download the Hub app from the Google Play store, introduce the suite of available mobile phone apps, build rapport, and set expectations for the coach-participant relationship. Thereafter, participants received 1-2 texts per week from their coach to provide support, offer encouragement, reinforce app use, and check-in on progress or challenges. Coaches also responded to all participant-initiated texts within 1 working day. Coaches were trained and monitored by one of the authors (KNT) and had at least a bachelor’s degree.

The coaches had a dashboard that provided information about the IntelliCare apps on each participant’s phone, including which apps were installed, when they were downloaded, each time an app was used, and which apps were selected as “primary” in the Hub app. The dashboard also included an short message service (SMS) messaging tool, a section for brief notes, and an alert indicating when no IntelliCare app had been used for 3 days, prompting coaches to check in.

Coaches were explicitly prohibited from making any recommendations about which apps to use, primarily because the aim of developing a recommender system required for that use and app selection reflect users’ intentions and actions. Thus, the major focus in the early part of the trial was on encouraging engagement with the IntelliCare system, but not influencing which app or how frequently any particular app was used. This balance proved difficult to manage, resulting in confusion among participants about how they should use the IntelliCare system and the role of coaches (this information came from participant feedback interviews used for quality improvement) and difficulty on the part of coaches in how to manage participants’ confusion. Minor clarifications and adjustments were made to coaching protocol to try to address these issues; however, approximately halfway through the field trial, a somewhat more substantive modification was made to address these concerns. The protocol was changed such that coaches made a clear recommendation during the engagement call that participants focus on learning 1 new app per week, and this was reinforced by checking via a text asking which app they selected (even though this was usually visible on the dashboard). Coaches were instructed to encourage participants to first review the recommended apps and then explore the list of apps on their own when deciding which app to focus on for the week. If the participant reported being unsure about which app to choose, or directly asked the coach for a recommendation, coaches were then able to provide suggestions for specific apps. A 10-minute phone call at week 4 was also offered to check in on participant experiences with utilization of the program and any relevant concerns with the coaching.

### Coaches

Coaches (N=4) had at least a bachelor’s degree in psychology or a related field. Coaches received a detailed coaching manual, as well as training in the principles of coaching and motivational interviewing strategies. Coaches were also required to use the IntelliCare apps daily over several weeks during the training period and encouraged to continue to use the apps to maintain fluency with various aspects of treatment. Coaches also received 30-60 minutes of individual supervision per week, and also attended weekly group supervision including ongoing training didactics. Training and supervision were provided by one of the authors (KNT), who also authored the coaching manual.

### Assessment and Measurement

At baseline, prospective participants were screened and characterized using the Mini International Neuropsychiatric Interview (MINI). The MINI [40], a structured interview, was administered over the telephone by trained clinical evaluators who were supervised by a PhD level psychologist. Demographic information was collected via a self-report survey. Questions about pharmacotherapy and psychotherapy use were added shortly after the trial began, and 2 participants were not administered these questions. This explains the different denominator used for these results. Depression and anxiety were measured at baseline, week 4, and week 8 using the PHQ-9 and GAD-7, respectively. All assessment data were collected and managed using REDCap electronic data capture tools hosted at Northwestern University [41].

App use data from app launch and use logs were collected passively. An app use session is defined as a sequence of user-initiated actions or events separated by less than 5 minutes between events. A new app launch is defined as a new activity after 5 min of no activity (we note that some apps have audio or video content that may last longer than 5 min, in which case the running content is counted as activity). The length of an app use session is the time from first launch or use of an app to the last event in a session.

### Statistical Analyses

Baseline demographic and clinical characteristics were reported as frequency and percent for categorical variables and median and interquartile range (IQR) for continuous variables. Outcome measures over time were reported as means and standard deviations. Baseline characteristics were compared between participants meeting preliminary screening criteria and participants ultimately enrolled using a 2-sample *t* test for continuous variables and a chi-square test for categorical variables. Linear mixed-effects models were used to evaluate continuous PHQ-9 and GAD-7 scores over time and associations between participant demographic characteristics. Generalized linear mixed-effects models were used to evaluate symptom remission (PHQ-9<10 and GAD-7<8) over the study period. Participants completing at least two follow-up assessments were included in the analysis of primary outcomes. Usability of the IntelliCare apps were reported using frequencies and means. Session frequency over the course of the study period was evaluated using generalized linear mixed-effects regression models assuming a normal distribution. Differences in total app use sessions and total app time across participant demographic characteristics were assessed using the nonparametric Wilcoxon rank-sum test. All analyses were performed using SAS, version 9.4 (Cary, NC, USA).

## Results

### Participants

**Figure 1:**
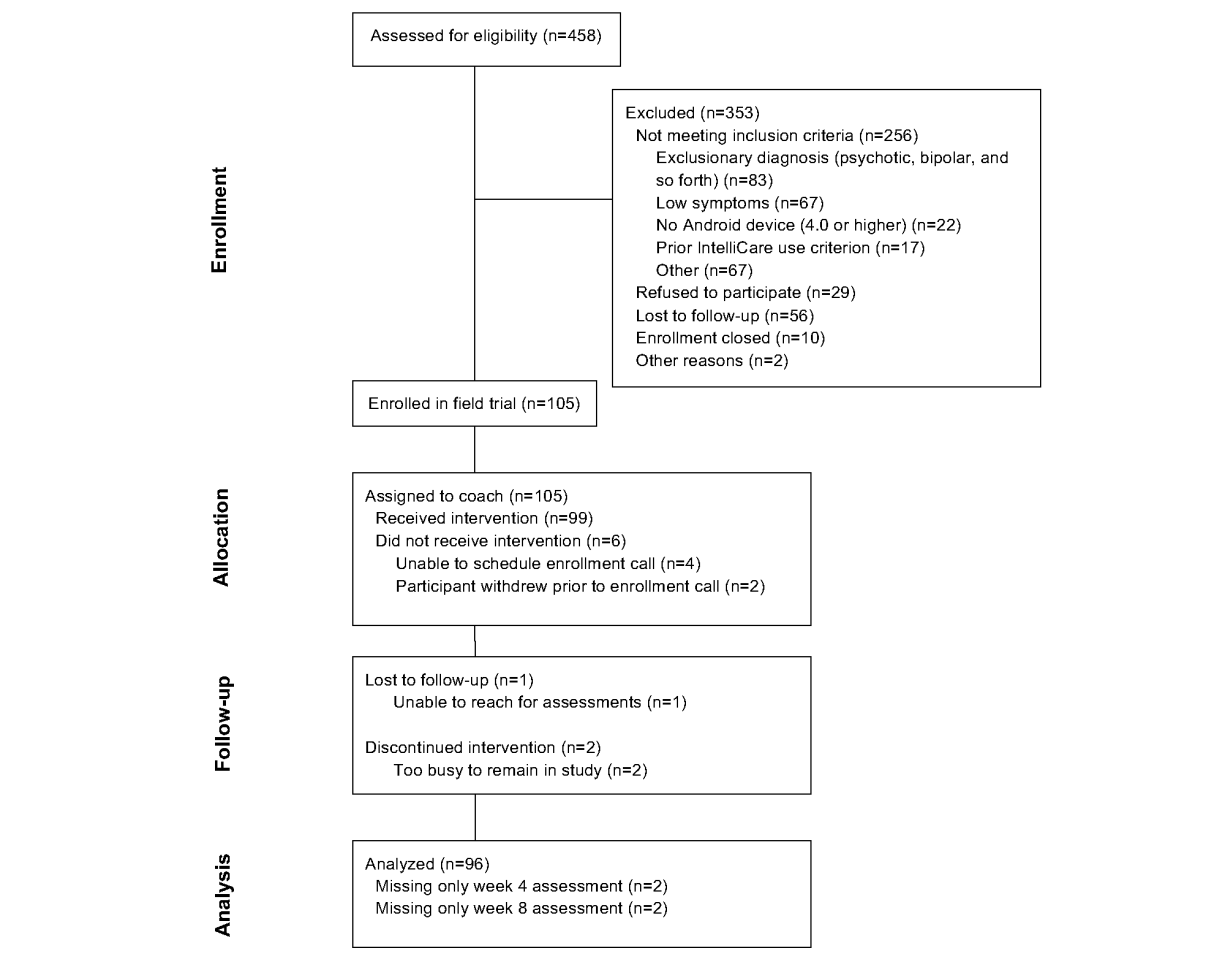
CONSORT Diagram of participant flow.

A total of 105 participants consented and were enrolled in the field trial. Of those enrolled, 6 participants did not respond to attempts to initiate treatment. An additional 3 participants did not complete any follow-up assessments after the baseline assessment, resulting in final analytic samples of 99 participants with usability data and 96 participants with at least two outcomes assessments. The flow of patients through the study is displayed in Figure 1.

Baseline characteristics of participants can be found in Table 2. The depression criterion of a PHQ-9 total score ≥10 was met by 78.1% (82/105) enrolled participants. The criterion for anxiety of a GAD-7 total score >8 was met also met by 78.1% (82/105) participants, and 60% (63/105) met criteria for both depression and anxiety.

**Table 2:** Sample characteristics (N=105).

Demographics		Median (IQR^a^) or n (%)
Age (years), median (IQR)		36 (27-50)
**Gender, n (%)**		
	Female	80 (76.2%)
	Male	25 (23.8%)
**Ethnicity, n (%)**		
	Not Hispanic or Latino	99 (94.3%)
	Hispanic or Latino	5 (4.8%)
	Declined to report	1 (1.0%)
**Race, n (%)**		
	Black or African American	8 (7.6%)
	American Indian or Alaska Native	1 (1.0%)
	Asian	6 (5.7%)
	White	88 (83.8%)
	More than 1 race	1 (1.0%)
	Declined to report	1 (1.0%)
**Marital status, n (%)**		
	Single	35 (33.3%)
	Married or domestic partner	38 (36.2%)
	Separated	1 (1.0%)
	Divorced	13 (12.4%)
	Widowed	3 (2.9%)
	Living with significant other	15 (14.3%)
**Education, n (%)**		
	Some high school	1 (1.0%)
	Completed high school or GED^b^	4 (3.8%)
	Some college	20 (19.1%)
	2-year college (Associate)	16 (15.2%)
	4-year college (BA, BS)	37 (35.2%)
	Master’s degree	22 (21.0%)
	Doctoral degree	4 (3.8%)
	Professional degree (MD, JD)	1 (1.0%)
**Employment status, n (%)**		
	Employed	76 (72.4%)
	Unemployed	11 (10.5%)
	Disability	8 (7.6%)
	Retired	5 (4.8%)
	Other	5 (4.8%)
Number of hours per week spent working, median (IQR)		40 (7-40)
**Income, median (IQR)**		
	Current total yearly household income	US $63,000 (30,000-100,000)
	Current total yearly personal gross income	US $35,000 (15,000-58,000)
**Treatment, n (%)**		
	Psychotherapy	23 (21.9%)
	Pharmacotherapy (n=103)	66 (64.1%)
**Recruitment source**		
	HealthPartners Healthcare System	38 (36.2%)
	Web or social media	16 (15.2%)
	ReearchMatch	11 (10.5%)
	Other	40 (38.1%)

^a^IQR: interquartile range.

^b^GED: general educational development.

**Table 3:** Means and standard deviations of outcome measures.

Outcome	Baseline	Week 4	Week 8	Pre or post effect size	*P* value
	n=96	n=94	n=94	(Cohen d)	
PHQ-9^a^, mean (SD)	12.5 (4.3)	8.4 (4.1)	6.4 (4.3)	1.4	<.001
GAD-7^b^, mean (SD)	10.9 (4.5)	7.1 (3.9)	5.8 (4.0)	1.2	<.001
PHQ-9 >10, n (%)	76 (79.2%)	36 (38.3%)	21 (22.3%)	N/A^c^	<.001
GAD-7 >8, n (%)	74 (77.1%)	42 (44.7%)	28 (29.8%)	N/A	<.001

^a^PHQ-9: Patient Health Questionnaire-9.

^b^GAD-7: Generalized Anxiety Disorder-7

^c^N/A: not applicable.

Pharmacotherapy was used by 64% (66/103) of participants, 22% (23/103) reported engaging in psychotherapy, (17% (18/105) reported using both pharmacotherapy and engaging in psychotherapy, and 30% (32/105) were untreated at baseline. There were no significant relationships between treatment status (pharmaco- or psychotherapy) and PHQ-9 or GAD-7 (*P*>.52). Among participants who met preliminary screening criteria (n=244), those who were enrolled (n=105) did not differ significantly from those not enrolled (n=139) on demographic data including age (*P*=.46), gender (*P*=.66), ethnicity (*P*=.55), race (*P*=.12), or score on PHQ-8 (*P*=.97) or GAD-7 (*P*=.96).

### Outcomes for Depression and Anxiety

Descriptive statistics for outcome measures over time are summarized in Table 3 for participants completing at least two assessments.

Significant improvements were seen across the entire sample for both PHQ-9 (*P*<.001) and GAD-7 (*P*<.001). Among participants meeting entry criteria for depression, significant reductions were present on both the PHQ-9 (*P* ≤.001) and the GAD-7 (*P* ≤.001). Similarly, among participants meeting entry criteria for anxiety, significant reductions were present on both the PHQ-9 (*P* ≤.001) and the GAD-7 (*P* ≤.001).

At the end of the treatment, 37% (35/105) of participants met criteria for full remission or no symptoms of depression (PHQ-9<5), 40% (38/105) met criteria for recovery or mild symptoms (PHQ-9=5-9), and 22% (21/105) continued to meet criteria for referral for treatment (PHQ-9≥10). Similarly, 42% (39/105) of participants met criteria for full remission or no symptoms of anxiety (GAD-7<5), 45% (42/105) met criteria for recovery or mild symptoms (GAD-7=5-9), and 14% (13/105) continued to meet criteria for referral for treatment (GAD-7≥10).

Neither baseline PHQ-9 nor GAD-7 were significantly related to changes in depression or anxiety severity, or concurrent treatment with psychotherapy or pharmacotherapy (*P* s>.19). There were no significant relationships between gender, education, ethnicity, or use of psychotherapy or antidepressants in either the PHQ-9 or GAD-7 outcomes (*P* s=.10-.79). There was a significant effect of age on GAD-7 total score, with greater age being associated with improved anxiety (*P*=.01), although age was not associated with PHQ-9 scores (*P*=.20).

### App Usage

Trial participants initiated an average of 191.4 (SD 139.2; median 180) IntelliCare treatment app sessions during the 8 weeks of the trial. Of the 99 participants who initiated the treatment, 96.0% (95/99) continued to use the apps at week 5 and 90.1% (90/99) continued through week 8. Table 4 reports the mean and median number of treatment app sessions by study week, which did not change significantly over time (*P*=.10).

**Table 4:** Mean number of treatment app use sessions by study week.

Week	n used	Mean (SD)	Median	Interquartile range	Minimum	Maximum
1	98	20.09 (15.63)	15	10-26	0	105
2	97	22.79 (16.76)	20	11-31	0	81
3	97	24.1 (20.21)	20	10-31	0	124
4	94	25.33 (20.68)	20	10-37	0	100
5	94	26.07 (20.41)	23	12-35	0	103
6	91	26.25 (23.66)	21	7-41	0	117
7	89	23.44 (22.27)	18	6-37	0	126
8	90	23.3 (25.57)	14	8-30	0	163

**Table 5:** Mean frequency of individual treatment app and Hub app use sessions.

App	n used	Mean (SD)	Median	Interquartile range	Minimum	Maximum
Day to Day	78	34.4 (48.3)	14	1-50	0	230
Daily Feats	73	25.8 (32.1)	17	0-39	0	217
Slumber Time	72	18.3 (21.3)	13	0-27	0	109
Purple Chill	81	17.8 (17.9)	13	3-28	0	77
My Mantra	62	15.4 (21.8)	6	0-26	0	100
Thought Challenger	82	13.4 (16.3)	8	2-17	0	70
Aspire	73	13.0 (15.2)	9	0-17	0	68
iCope	70	11.7 (20.6)	4	0-11	0	125
Boost Me	68	10.1 (11.1)	7	0-14	0	47
Move Me	74	9.9 (12.8)	5	0-14	0	67
Worry Knot	74	9.2 (10.6)	7	0-12	0	55
Social Force	56	6.9 (11.1)	2	0-9	0	60
Me Locate	51	5.5 (8.5)	1	0-9	0	42
Total	99	195.4 (141)	182	95-263	9	844
Hub app	99	107.32 (71.29)	92	51-152	6	372

The average length of use for each session was 1.4 min (SD 3.9; median 18 seconds). The Hub app was launched an average of 107.3 (SD 71.3; median 92) times over the 8 weeks.

There were no significant relationships between age, education, employment status, gender, race, or ethnicity and the number of treatment app sessions or the length of time spent in the apps (*P*=.18-.68). There were significant effects of age, gender, and antidepressant medication on total time spent using the apps. Older patients tended to use the apps for a longer time than younger patients (*P*=.003), as did women (*P*=.03) and participants using medications (*P*=.01). Additionally, there was a marginal effect for engagement in individual psychotherapy (*P*=.055) or pharmacotherapy (*P*=.054) with those in treatment spending more time in the apps.

Table 5 shows the mean frequency of individual treatment app and Hub app use sessions over the 8 weeks of treatment.

Eight (8.1%) participants downloaded 5 or fewer of the IntelliCare Treatment apps, 60 (60.6%) downloaded between 6 and 10 apps, and 31 (31.3%) downloaded 11-13 apps. The Hub app was launched at least six times by all participants who initiated treatment over the study period.

### Coaching

Among the 99 participants who initiated treatment, 97% (96/99) continued to participate in coaching at week 5, and 93% (92/99) continued through the end of the trial. Because some texts were broken into more than 1 SMS transmission, we report here on days when texts were sent, rather than number of texts, to avoid inflating results. On average, coaches sent texts to participants on 22.2 (SD 4.7) study days (39.6%) over the 8 weeks of treatment. Participants sent texts to their coach on an average of 16.7 (SD 5.8) study days (29.9%). All 99 participants who initiated treatment received an engagement call, lasting on average 39.1 (SD 7.7) minutes. The first 34 participants did not receive an offer for a midpoint follow-up call. In response to participant feedback interviews indicating some confusion around procedures, expectations, and the role of the coach, a second call was offered to participants beginning with the 35th person enrolled, which lasted on average 12.8 (SD 8.5) minutes.

After the change in the coaching protocol, participants no longer commented on confusion surrounding the role of coaches. There was no significant difference in outcomes on the PHQ-9, GAD-7, number of app sessions, or total time on IntelliCare apps between participants receiving the initial and revised coaching protocols (*P=*.33-.90).

### App Use Before and After the Trial

Because IntelliCare apps were freely available on the Google Play Store, we examined the number of people who had used apps prior to enrolling in the study. Of the 99 who initiated treatment, 33.3% (33/99) had used at least one of the apps for an average of 16.6 days (SD 11.2; median 15) prior to enrollment. There was no significant difference in outcomes on the PHQ-9, GAD-7, number of app sessions, or time using apps (*P*=.29-.93) between those with prior exposure to the IntelliCare apps and those without.

After completion of the study, 33.7% (29/99) people continued to use the IntelliCare apps 2 weeks later, 20.9% (18/99) at 1 month, 22% (19/99) between months 1 and 2, and 23.2% (20/99) beyond 2 months.

### Safety

There were no adverse events (eg, suicide attempts, psychiatric hospitalizations).

## Discussion

### Principal Findings

In this pilot feasibility study, the IntelliCare apps and coaching showed large reductions in symptoms of depression and anxiety. These improvements were largely consistent across many demographic variables, although outcomes were slightly worse for those who reported receiving disability slightly better for older participants. Outcomes were unrelated to whether or not the participant was receiving psychotherapy or antidepressant medications during the treatment.

App usage was substantial, with an average of 195 app launches per participant over the course of 8 weeks of treatment. This is considerably higher than has been reported for other apps that have ranged on the order of 15-22 launches in total [21,42]. There are several likely reasons for this. First, the design of the IntelliCare suite is markedly different from other apps. Most of the IntelliCare apps are intended to be used frequently and briefly; other apps that have been described in the literature do not necessarily have the same frequency expectations. In addition, IntelliCare is a suite of apps, in which people are expected and encouraged to swap apps in and out of their use rotation, thereby maintaining novelty and engagement. Finally, while the use statistics for IntelliCare apps downloaded “in the wild,” without a coach or participation in a study, were also much higher than is typically seen [28], use statistics in this study were far higher than even those, which was likely the result of our use of low-intensity coaching. Given coaching required only around 40-50 min of call time per subject, along with a small amount of time for composing and sending text messages, IntelliCare has the potential to be very cost-effective.

### Novel Treatment Design

The approach used here differs substantially from previous mHealth and eHealth interventions for depression or anxiety in several ways. First, IntelliCare apps are very interactive, emphasizing the application of skills through in-app actions. Most eHealth interventions for depression or anxiety have used psychoeducational approaches, and mobile phone approaches have tended to use the phone primarily for symptom and mood monitoring, leaving primary treatment to clinicians or websites [20,21,43]. These interventions attempt to provide understanding for the user as part of an effort to persuade the person to engage in a new treatment behavior (eg, engaging in positive behaviors or thought restructuring). The IntelliCare apps place the action (eg, goal setting, checklists, reminders, logging, and so forth) at the beginning of the interaction. Most IntelliCare apps have only limited explanation of why the action would be useful, most of which is relegated to the Help section. Thus, users download the app and begin with the exercise, not the explanation. Essentially, IntelliCare operates from the assumption that doing is learning. Understanding will come from doing, and it is not necessary that everyone have the same understanding for the exercise to be useful.

The interactions supporting skills training were designed to be brief and frequent. Given there is little to no explanation, interactions are designed to be as intuitive as possible. With mean app session lengths of 1 minute, median app session rates of 17 seconds, and mean weekly app use frequencies of 21-29, with no drop-off over time, that goal appears to have been achieved.

IntelliCare was designed to be eclectic. Apps draw from a variety of theoretical orientations, and users are encouraged to identify apps they perceive as useful and consistent with their goals rather than following a particular conceptual model. Indeed, 95% of participants used 5 or more apps during the study, supporting the notion that users are willing to use multiple apps even when they are not necessarily organized by a therapy orientation. In this way, approaches that construct metaphorical toolboxes through digital tools might be a good way to present potential options and allow individual users to tailor treatment and select options that fit their own interests and need, for example, [44,45]. Furthermore, the Hub app was used frequently, supporting the idea that that apps or interfaces that help organize these toolboxes might help improve user experience across such apps.

### Future Research

A longer-term goal for IntelliCare is to develop a recommender system that will be able to use passively collected data, such as app usage, to identify and recommend apps that are likely to be acceptable and useful to the individual. Thus, while the recommendations offered through the Hub app in this study were random, the Hub app is intended to provide recommendations that are of value to the user. Rather than basing such recommendations on rules derived from a psychological orientation, recommendations will be based on accumulating knowledge from the entire population of app users. Such a system could be modifiable and extensible, identifying apps that are underperforming so that they can be removed from the system and accommodate the introduction of new apps. Thus, while the focus of this study has been largely on the apps within the IntelliCare system, the longer-term vision for IntelliCare is as a platform that can use available data acquired from the suite of apps to monitor efficacy and provide evidence-based recommendations. The theoretical underpinnings for such a system have been described elsewhere [46] and are the focus of ongoing research.

### Coaching Protocol

This field trial used a coaching protocol aimed at encouraging efficient use of the apps [39,47]. This protocol was developed with 2 aims in mind. First, the literature generally suggests that human support enhances adherence and perhaps outcomes for digital interventions [10]. Our open deployment on Google Play suggested that, compared with many health apps [38], IntelliCare apps appear to be relatively engaging, with mean number of uses ranging from 3.1 to 17.0, and mean time from first to last launch ranging from 13.0 to 25.3 days, depending on the app [28]. This field trial found use rates that far exceeded those rates; however, it is difficult to compare these 2 sets of findings because the Google Play deployment analyzed data from anyone who downloaded the app, while this study selected participants with significant symptoms and sufficient motivation to be in a research study. Additionally, the coaching protocol aimed to ensure sufficient use of a variety of the apps that could help support analytics necessary to develop a recommendation engine.

It is interesting to note that changes made to the coaching protocol during this field trial had no observable impact on symptoms or app usage. This is notable because the user feedback interviews conducted for quality improvement at the end of the study indicated that participants were much clearer about the role of the coaches after this change in the coaching protocol. This impression was also mirrored by greater clarity among coaches in their role.

Finally, this is the first study we are aware of that has evaluated a freely available digital intervention in a coached form, and thus shed light on the relationship between digitally enabled services and the apps or tools available on app stores that support them. About a third of participants had prior exposure to the apps, and a third continued to use them after the end of coaching. Those who used apps prior to engaging with the study and treatment may have been evaluating the apps before committing to the treatment and study, and indeed some people found their way to the study through the apps. This suggests that apps may serve both as an initial point of evaluation for potential patients, as well as a conduit through which services can be provided. This is very consistent with marketing practices for digitally enabled services generally, and apps and websites designed for the possibility of both self-guided and human-supported consumer experiences that are becoming increasingly more frequent in the mental health space.

### Limitations

A number of limitations should be noted in considering these results. First, because this was a single-arm trial, we cannot rule out the possibility that the improvement in depression and anxiety, although impressive, was due to factors other than IntelliCare. For example, it is possible that we recruited a sample that was likely to improve anyway. Second, if the IntelliCare system did in fact have a positive impact, we cannot disentangle the effects of the coaching and the apps. Third, the coaching protocol was not constructed under ideal circumstances, as coaches’ role encouraging the use of the overall system conflicted in practice with the prohibition from influencing participants’ use of specific apps. Fourth, it is possible that study compensation contributed to treatment adherence. We believe this is unlikely, given participants were clearly informed that compensation was for completion of assessments only, and the university’s payment processing system results in delays of up to 2 months for payments. Finally, the apps are currently available on Android only, and this user base and their use of apps might be different from users of other platforms. Our team plans iOS development, which will address this shortcoming.

### Conclusions

These shortcomings notwithstanding, this study showed clear support for a novel mHealth system for providing mental health treatment. Although other groups, such as the National Center for Telehealth and Technology, have developed multiple mental health apps covering diverse treatment strategies, targets, and populations (eg, Breathe2Relax, CBT-i Coach, Moving Forward, Positive Activity Jackpot, PTSD Coach, T2 Mood Tracker), IntelliCare represents the first effort to make a unified, consolidated app experience. Novel features of the IntelliCare system include that it is elemental, allowing individual apps to be used or not used based on their effectiveness and utility and it is eclectic, viewing treatment strategies as elements that can be applied as needed rather than based on 1 theoretical model. The future of mHealth is likely not to rest on a singular approach, or a singular app. Therefore, it is necessary to consider platforms that can consolidate efforts across a variety of apps such as IntelliCare.

## Abbreviations

BIT: behavioral intervention technology
GAD-7: Generalized Anxiety Disorder-7
IQR: interquartile range
MINI: Mini International Neuropsychiatric Interview
PHQ-9: Patient Health Questionnaire-9
SMS: short message service

## Appendix

Multimedia Appendix 1 IntelliCare: Selected Annotated Screenshots.

## References

[1] Cuijpers P, van SA, Andersson G, van OP (2008). Psychotherapy for depression in adults: a meta-analysis of comparative outcome studies. J Consult Clin Psychol.

[2] Hofmann SG, Smits JA (2008). Cognitive-behavioral therapy for adult anxiety disorders: a meta-analysis of randomized placebo-controlled trials. J Clin Psychiatry.

[3] Dwight-Johnson M, Sherbourne CD, Liao D, Wells KB (2000). Treatment preferences among depressed primary care patients. J Gen Intern Med.

[4] Bedi N, Chilvers C, Churchill R, Dewey M, Duggan C, Fielding K, Gretton V, Miller P, Harrison G, Lee A, Williams I (2000). Assessing effectiveness of treatment of depression in primary care. Partially randomised preference trial. Br J Psychiatry.

[5] Churchill R, Khaira M, Gretton V, Chilvers C, Dewey M, Duggan C, Lee A, Nottingham CounsellingAntidepressants in Primary Care (CAPC) Study Group (2000). Treating depression in general practice: factors affecting patients' treatment preferences. Br J Gen Pract.

[6] Priest RG, Vize C, Roberts A, Roberts M, Tylee A (1996). Lay people's attitudes to treatment of depression: results of opinion poll for Defeat Depression Campaign just before its launch. BMJ.

[7] Mohr DC, Ho J, Duffecy J, Baron KG, Lehman KA, Jin L, Reifler D (2010). Perceived barriers to psychological treatments and their relationship to depression. J Clin Psychol.

[8] Mohr DC, Hart SL, Howard I, Julian L, Vella L, Catledge C, Feldman MD (2006). Barriers to psychotherapy among depressed and nondepressed primary care patients. Ann Behav Med.

[9] Kazdin A, Blase S (2011). Rebooting Psychotherapy Research and Practice to Reduce the Burden of Mental Illness. Perspect Psychol Sci.

[10] Richards D, Richardson T (2012). Computer-based psychological treatments for depression: a systematic review and meta-analysis. Clin Psychol Rev.

[11] Arnberg FK, Linton SJ, Hultcrantz M, Heintz E, Jonsson U (2014). Internet-delivered psychological treatments for mood and anxiety disorders: a systematic review of their efficacy, safety, and cost-effectiveness. PLoS One.

[12] Renton T, Tang H, Ennis N, Cusimano MD, Bhalerao S, Schweizer TA, Topolovec-Vranic J (2014). Web-based intervention programs for depression: a scoping review and evaluation. J Med Internet Res.

[13] Torous J, Powell A (2015). Current research and trends in the use of smartphone applications for mood disorders. Internet Interventions.

[14] Pew Research Center.

[15] Krebs P, Duncan DT (2015). Health App Use Among US Mobile Phone Owners: A National Survey. JMIR Mhealth Uhealth.

[16] Torous J, Friedman R, Keshavan M (2014). Smartphone ownership and interest in mobile applications to monitor symptoms of mental health conditions. JMIR Mhealth Uhealth.

[17] Watts S, Mackenzie A, Thomas C, Griskaitis A, Mewton L, Williams A, Andrews G (2013). CBT for depression: a pilot RCT comparing mobile phone vs. computer. BMC Psychiatry.

[18] Oulasvirta A, Tamminen S, Roto V, Kuorelahti J (2005). Interaction in 4-Second Bursts: The Fragmented Nature of Attentional Resources in Mobile HCI.

[19] Vaish R, Wyngarden K, Chen J, Cheung B, Bernstein M (2014). Twitch Crowdsourcing: Crowd Contributions in Short Bursts of Time.

[20] Donker T, Petrie K, Proudfoot J, Clarke J, Birch M, Christensen H (2013). Smartphones for smarter delivery of mental health programs: a systematic review. J Med Internet Res.

[21] Proudfoot J, Clarke J, Birch M, Whitton AE, Parker G, Manicavasagar V, Harrison V, Christensen H, Hadzi-Pavlovic D (2013). Impact of a mobile phone and web program on symptom and functional outcomes for people with mild-to-moderate depression, anxiety and stress: a randomised controlled trial. BMC Psychiatry.

[22] Huguet A, Rao S, McGrath PJ, Wozney L, Wheaton M, Conrod J, Rozario S (2016). A Systematic Review of Cognitive Behavioral Therapy and Behavioral Activation Apps for Depression. PLoS One.

[23] Schueller SM, Munoz RF, Mohr DC (2013). Realizing the Potential of Behavioral Intervention Technologies. Curr Dir Psychol Sci.

[24] Mohr DC, Schueller SM, Montague E, Burns MN, Rashidi P (2014). The behavioral intervention technology model: an integrated conceptual and technological framework for eHealth and mHealth interventions. J Med Internet Res.

[25] Institute of Medicine (IOM) (2015). Psychosocial Interventions for Mental and Substance Use Disorders: A Framework for Establishing Evidence-Based Standards.

[26] Beutler LE, Harwood MT (2000). Prescriptive psychotherapy: A practical guide to systemic treatment selection.

[27] Chorpita BF, Daleiden EL (2009). Mapping evidence-based treatments for children and adolescents: application of the distillation and matching model to 615 treatments from 322 randomized trials. J Consult Clin Psychol.

[28] Lattie EG, Schueller SM, Sargent E, Stiles-Shields C, Tomasino KN, Corden ME, Begale M, Karr CJ, Mohr DC (2016). Uptake and Usage of IntelliCare: A Publicly Available Suite of Mental Health and Well-Being Apps. Internet Interv.

[29] Stiles-Shields C, Montague E, Mohr DC (2016). The use of scenario-based design for the development of behavioral intervention technologies.

[30] Versluis A, Verkuil B, Spinhoven P, van der Ploeg MM, Brosschot JF (2016). Changing Mental Health and Positive Psychological Well-Being Using Ecological Momentary Interventions: A Systematic Review and Meta-analysis. J Med Internet Res.

[31] Andersson G, Bergström J, Buhrman M, Carlbring P, Holländare F, Kaldo V, Nilsson-Ihrfelt E, Paxling B, Ström L, Waara J (2008). Development of a New Approach to Guided Self-Help via the Internet: The Swedish Experience. J Technol Hum Serv.

[32] Titov N, Dear BF, Staples LG, Bennett-Levy J, Klein B, Rapee RM, Shann C, Richards D, Andersson G, Ritterband L, Purtell C, Bezuidenhout G, Johnston L, Nielssen OB (2015). MindSpot Clinic: An Accessible, Efficient, and Effective Online Treatment Service for Anxiety and Depression. Psychiatr Serv.

[33] Kroenke K, Spitzer RL, Williams JB (2001). The PHQ-9: validity of a brief depression severity measure. J Gen Intern Med.

[34] Spitzer RL, Kroenke K, Williams JB, Löwe B (2006). A brief measure for assessing generalized anxiety disorder: the GAD-7. Arch Intern Med.

[35] Arroll B, Goodyear-Smith F, Crengle S, Gunn J, Kerse N, Fishman T, Falloon K, Hatcher S (2010). Validation of PHQ-2 and PHQ-9 to screen for major depression in the primary care population. Ann Fam Med.

[36] Löwe B, Decker O, Müller S, Brähler E, Schellberg D, Herzog W, Herzberg PY (2008). Validation and standardization of the Generalized Anxiety Disorder Screener (GAD-7) in the general population. Med Care.

[37] Martin A, Rief W, Klaiberg A, Braehler E (2006). Validity of the Brief Patient Health Questionnaire Mood Scale (PHQ-9) in the general population. Gen Hosp Psychiatry.

[38] Helander E, Kaipainen K, Korhonen I, Wansink B (2014). Factors related to sustained use of a free mobile app for dietary self-monitoring with photography and peer feedback: retrospective cohort study. J Med Internet Res.

[39] Mohr DC, Cuijpers P, Lehman K (2011). Supportive accountability: a model for providing human support to enhance adherence to eHealth interventions. J Med Internet Res.

[40] Sheehan DV, Lecrubier Y, Sheehan KH, Amorim P, Janavs J, Weiller E, Hergueta T, Baker R, Dunbar GC (1998). The Mini-International Neuropsychiatric Interview (M.I.N.I.): the development and validation of a structured diagnostic psychiatric interview for DSM-IV and ICD-10. J Clin Psychiatry.

[41] Harris PA, Taylor R, Thielke R, Payne J, Gonzalez N, Conde JG (2009). Research electronic data capture (REDCap)--a metadata-driven methodology and workflow process for providing translational research informatics support. J Biomed Inform.

[42] Roepke AM, Jaffee SR, Riffle OM, McGonigal J, Broome R, Maxwell B (2015). Randomized Controlled Trial of SuperBetter, a Smartphone-Based/Internet-Based Self-Help Tool to Reduce Depressive Symptoms. Games Health J.

[43] Shen N, Levitan M, Johnson A, Bender JL, Hamilton-Page M, Jadad AA, Wiljer D (2015). Finding a depression app: a review and content analysis of the depression app marketplace. JMIR Mhealth Uhealth.

[44] Parks AC, Della PM, Pierce RS, Zilca R, Lyubomirsky S (2012). Pursuing happiness in everyday life: the characteristics and behaviors of online happiness seekers. Emotion.

[45] Schueller SM, Leykin Y, Pérez-Stable EJ, Muñoz RF (2013). Selection of intervention components in an internet stop smoking participant preference trial: beyond randomized controlled trials. Psychiatry Res.

[46] Mohr DC, Cheung K, Schueller SM, Hendricks BC, Duan N (2013). Continuous evaluation of evolving behavioral intervention technologies. Am J Prev Med.

[47] Schueller S, Tomasino K, Mohr D (2016). Integrating Human Support into Behavioral Intervention Technologies: The Efficiency Model Clinical Psychology: Science and Practice. Clin Psychol Sci Prac.

