# Supplementary material for: IntelliCare: An Eclectic, Skills-Based App Suite for the Treatment of Depression and Anxiety
Source: J Med Internet Res. 2017 Jan 5;19(1):e10. doi: 10.2196/jmir.6645 (PMC5247623; doi:10.2196/jmir.6645)

Supplement

Sample Screenshots from Six of the Patient-Facing Apps and from Coordinator Dashboards

IntelliCare Hub

Manages messages and notifications from the other apps within the IntelliCare collection.

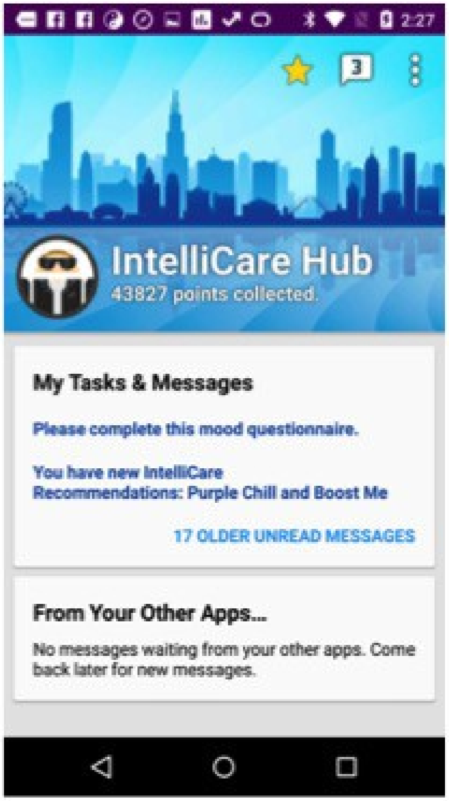

App home screen consolidates messages from all IntelliCare apps.

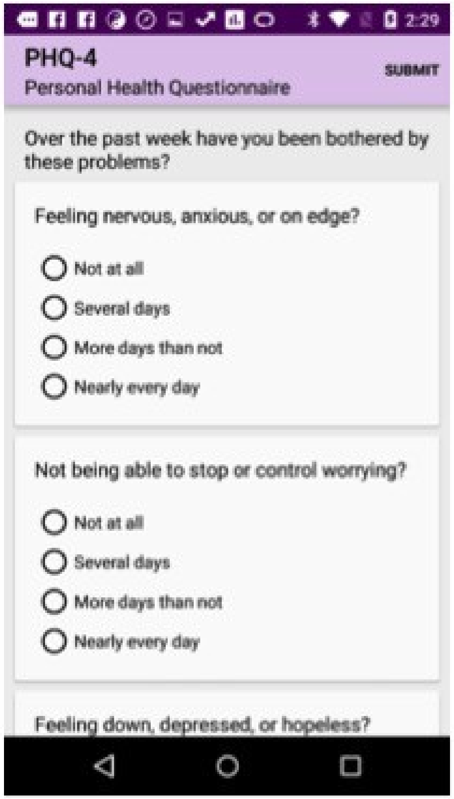

Users are prompted to complete short symptom assessments each week to alert their care team.

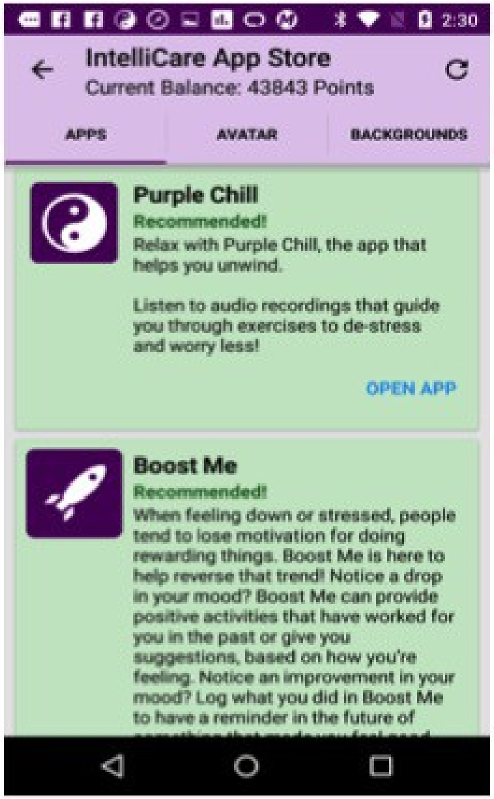

Users receive weekly recommendations for other IntelliCare apps likely to be useful to them, which can be downloaded directly through the Hub.

Daily Feats
Encourages the user to incorporate worthwhile and productive activities into the day. Users add accomplishments to the Feats calendar, where they can track their positive activity streaks and level up by completing more tasks.

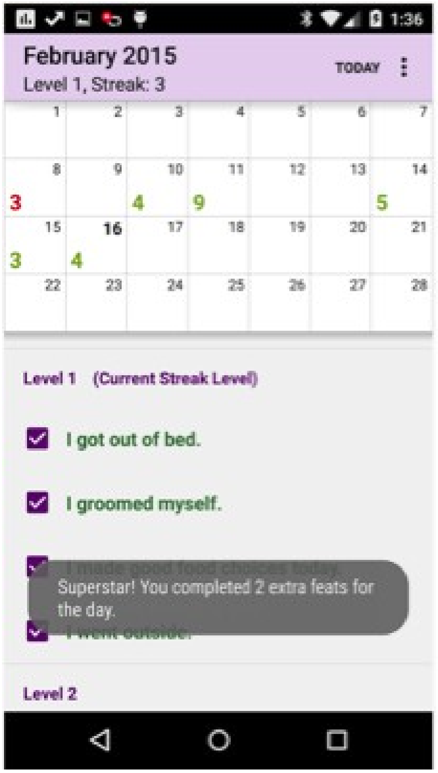

On download, user answers a few questions about their mood. Users are given 4 mood-appropriate “feats” to check off each day.

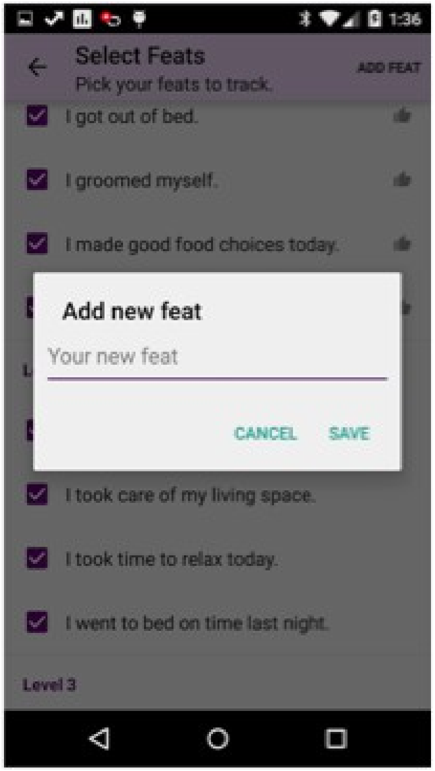

Users are given the option to add their own personal activity goals to their list

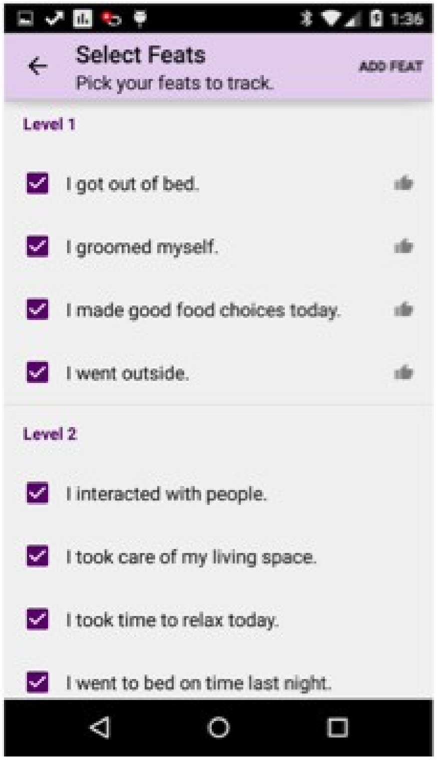

When users complete a streak (by completing more than 2 feats at their current level for 5 consecutive days), they are moved to the next level.

My Mantra

Prompts the user to create mantras (or repeatable phrases that highlight personal photo strengths and values and can motivate one to do and feel good) and construct virtual albums to serve as encouragement and reminders of these mantras.

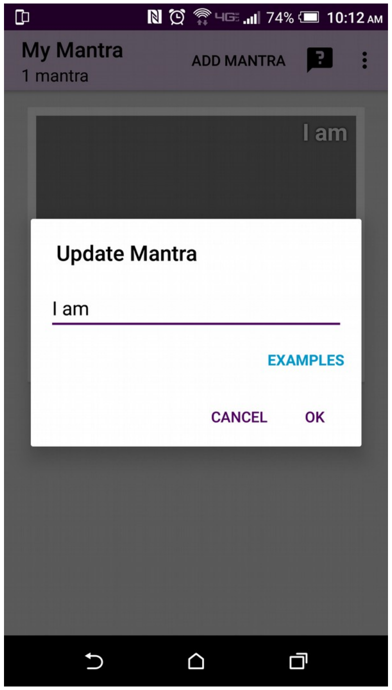

Users are prompted to enter a mantra upon opening the app

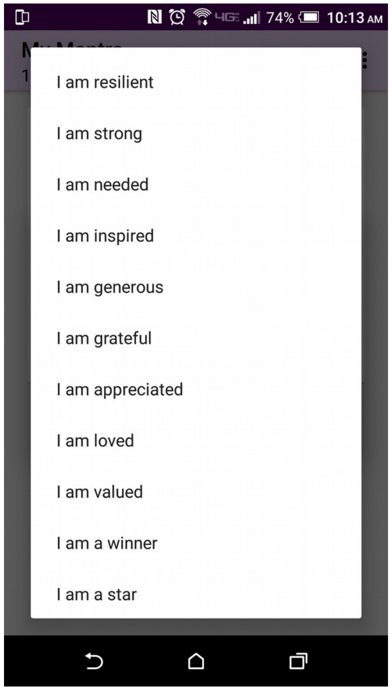

To get users started, a list of examples is provided

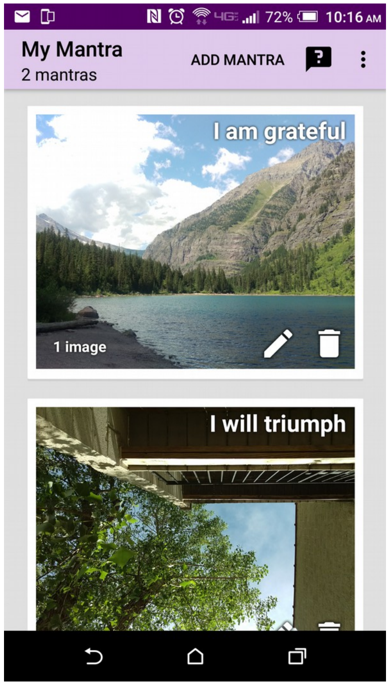

After a mantra is created, the app prompts the user to add a photo. Previously entered mantras and their associated pictures appear on the home screen

Purple Chill

Provides users with a library of audio recordings to relax and unwind. Teaches a variety of relaxation and mindfulness practices to de-stress and worry less.

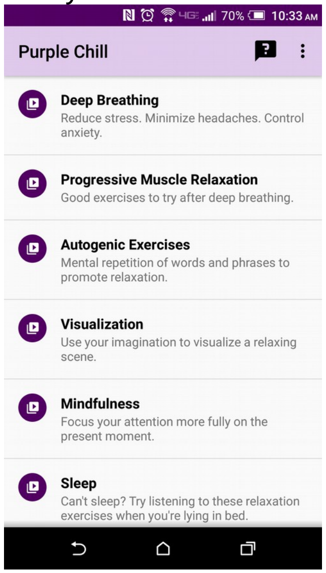

The home page displays groups of relaxation tracks organized by relaxation technique

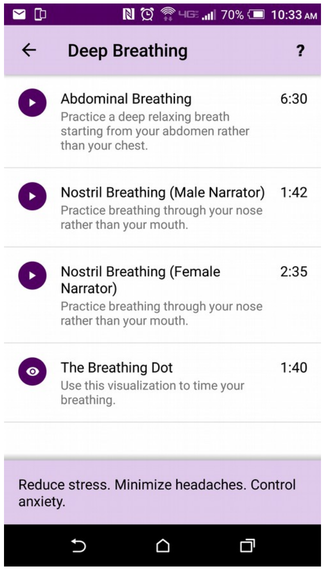

Users are provided with brief information about the nature and length of the track

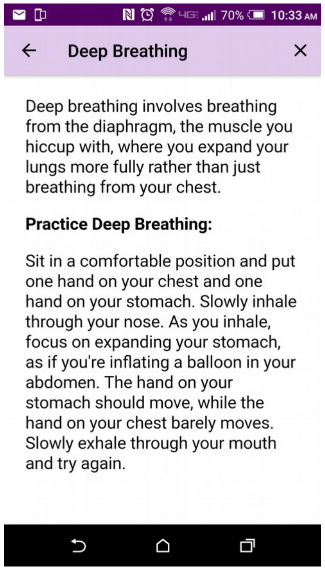

There is an introduction to each group of tracks that provides additional information about the technique

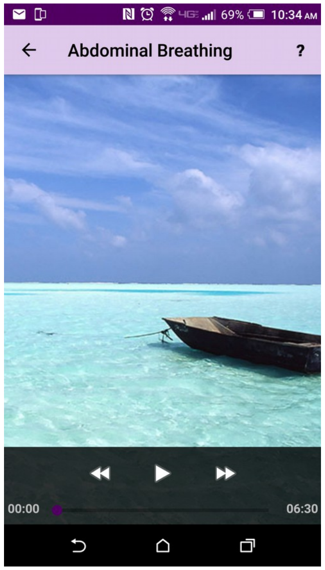

Audio tracks are paired with a relaxing visual image and can be paused

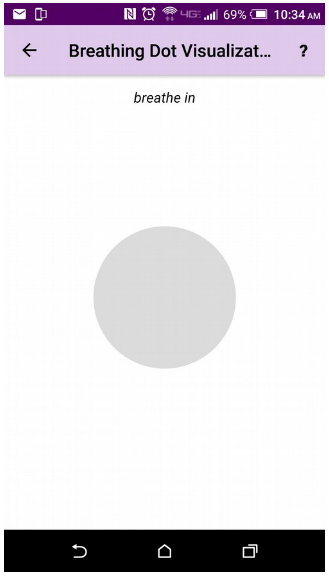

Visualizations are provided, such as the breathing dot in which the dot expands and retracts to guide breathing

Slumber Time

Prompts the user to complete sleep diaries to track sleep. Provides a bedtime checklist intended to clear one's mind before going to sleep. Provides audio recordings to facilitate rest and relaxation. Features an alarm clock function.

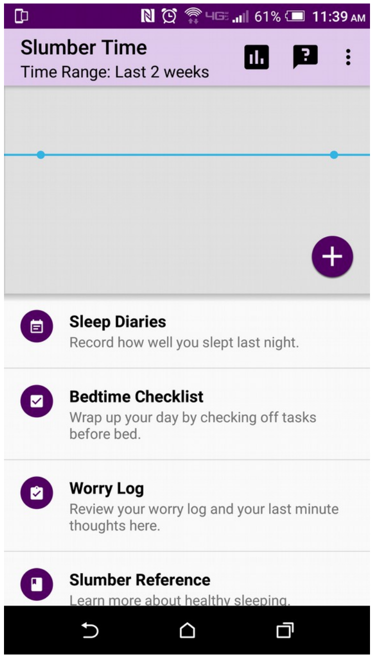

The home screen provides a graph of recently entered sleep data and allows the user to select different features of the app

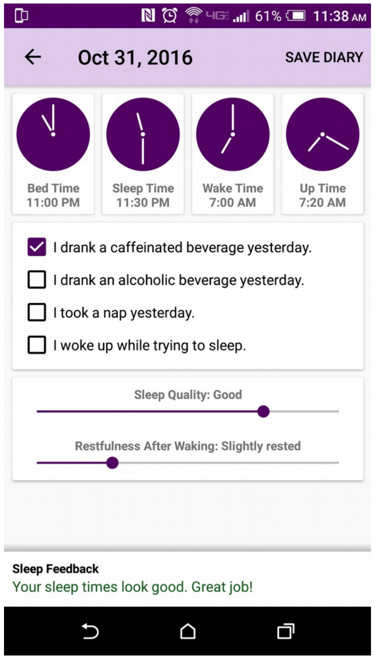

Users are prompted to complete a sleep diary each day and receive automated feedback

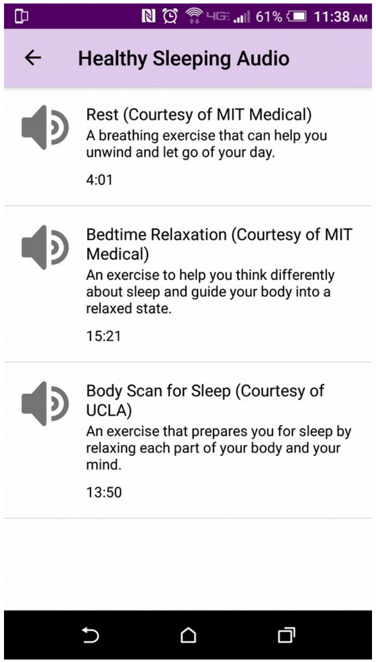

Users can access a library of audio tracks intending to prepare the user for sleep

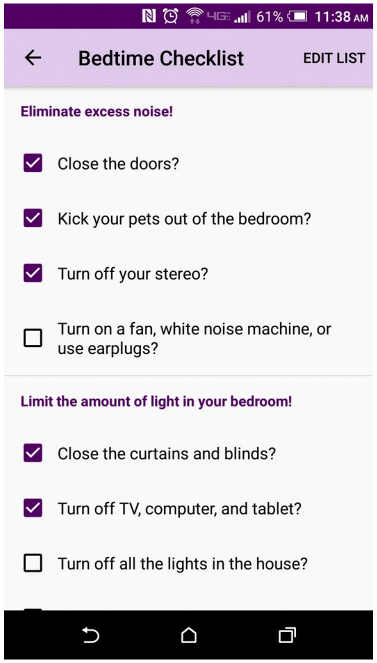

The bedtime checklist includes preprogrammed items and allows the user to add new, original items

Thought Challenger
A more traditional app that guides the user through an interactive cognitive restructuring tool to examine thoughts that might exaggerate negative experiences, lead one to be overcritical and bring down one's mood. Teaches the user to get into the habit of changing perspective and moving toward a more balanced outlook on life.

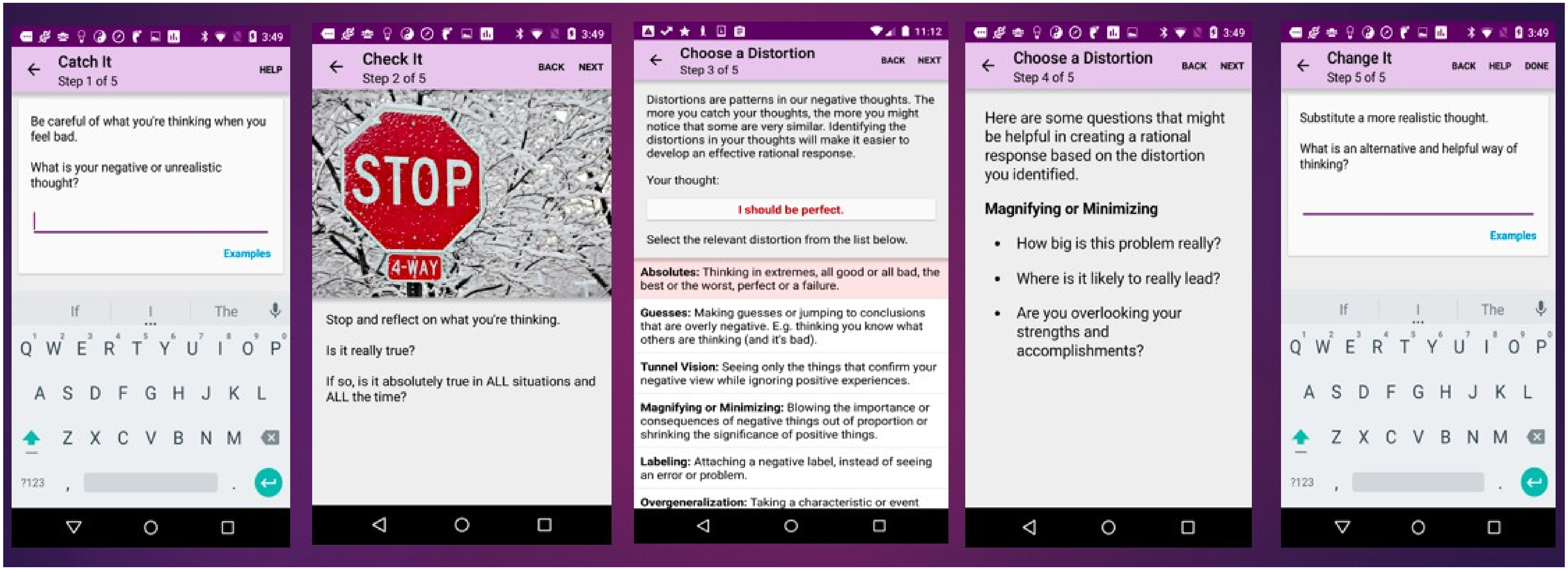

To begin the thought challenging process, users are prompted to identify a specific [illegible]

Next, users are prompted to question the validity of their [illegible]

Users view information on different types of cognitive distortions, a[illegible] select the relevant type

Users are prompted with distortion-specific questions to help them create an alternative thought

Finally, users are prompted to enter an alternative thought and can access examples to help guide them

IntelliCare Coach Dashboard

Our IntelliCare coach dashboard houses the text messaging interface and displays patient engagement with the app suite, including which apps are being used and when. The dashboard also includes alerts to support efficient triage and clinical decision-making and patients total score on brief symptom questionnaire (PHQ-4). Due to research protocols, this dashboard does not contain content from the apps (e.g. data from text fields as displayed below), which would be added in the proposed study.

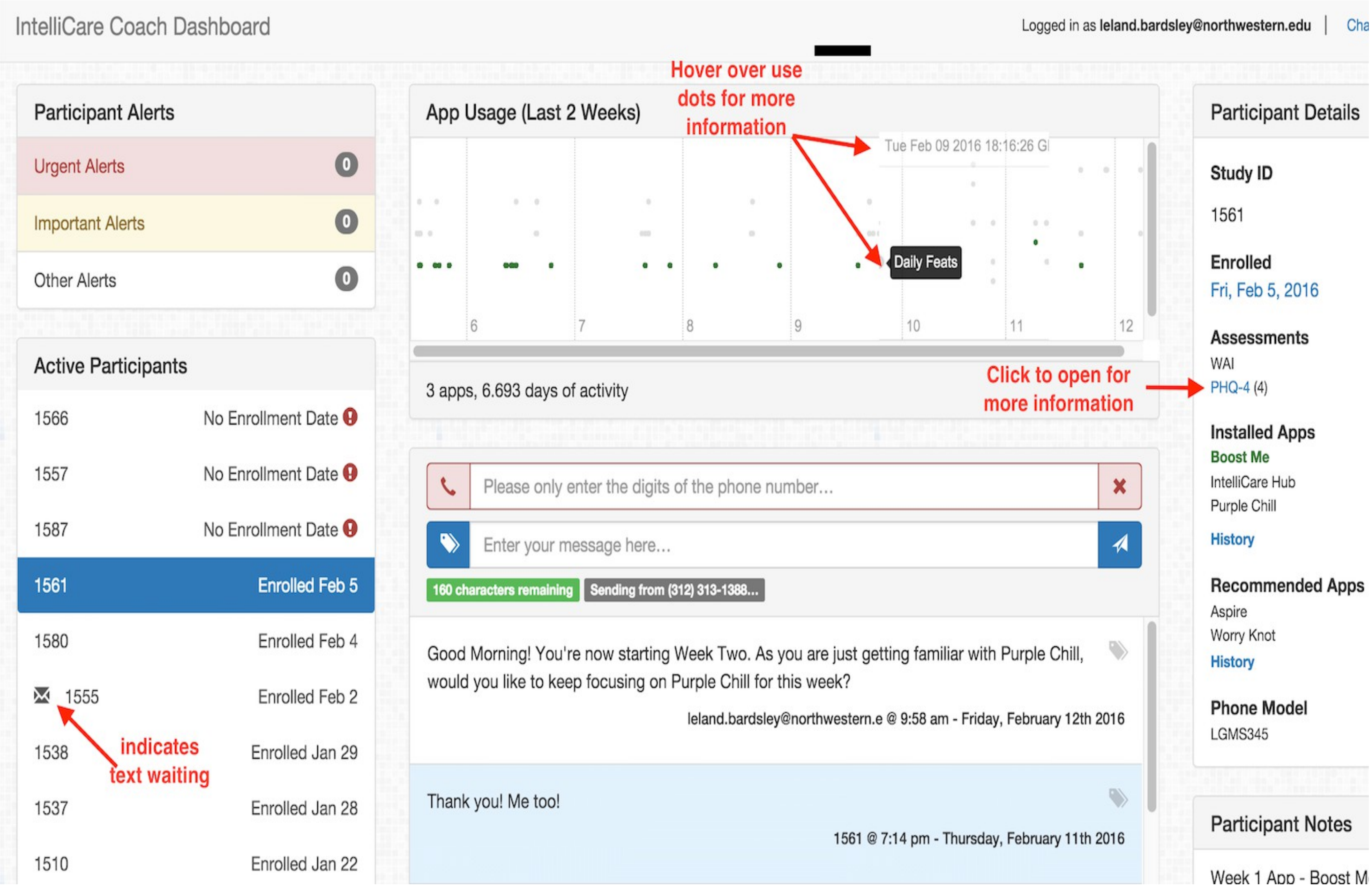

IntelliCare Coach Dashboard (continued)

A pop-up window allows coaches to view PHQ-4 responses in more detail and includes individual responses, prior scores, and a graphical representation of change over time.

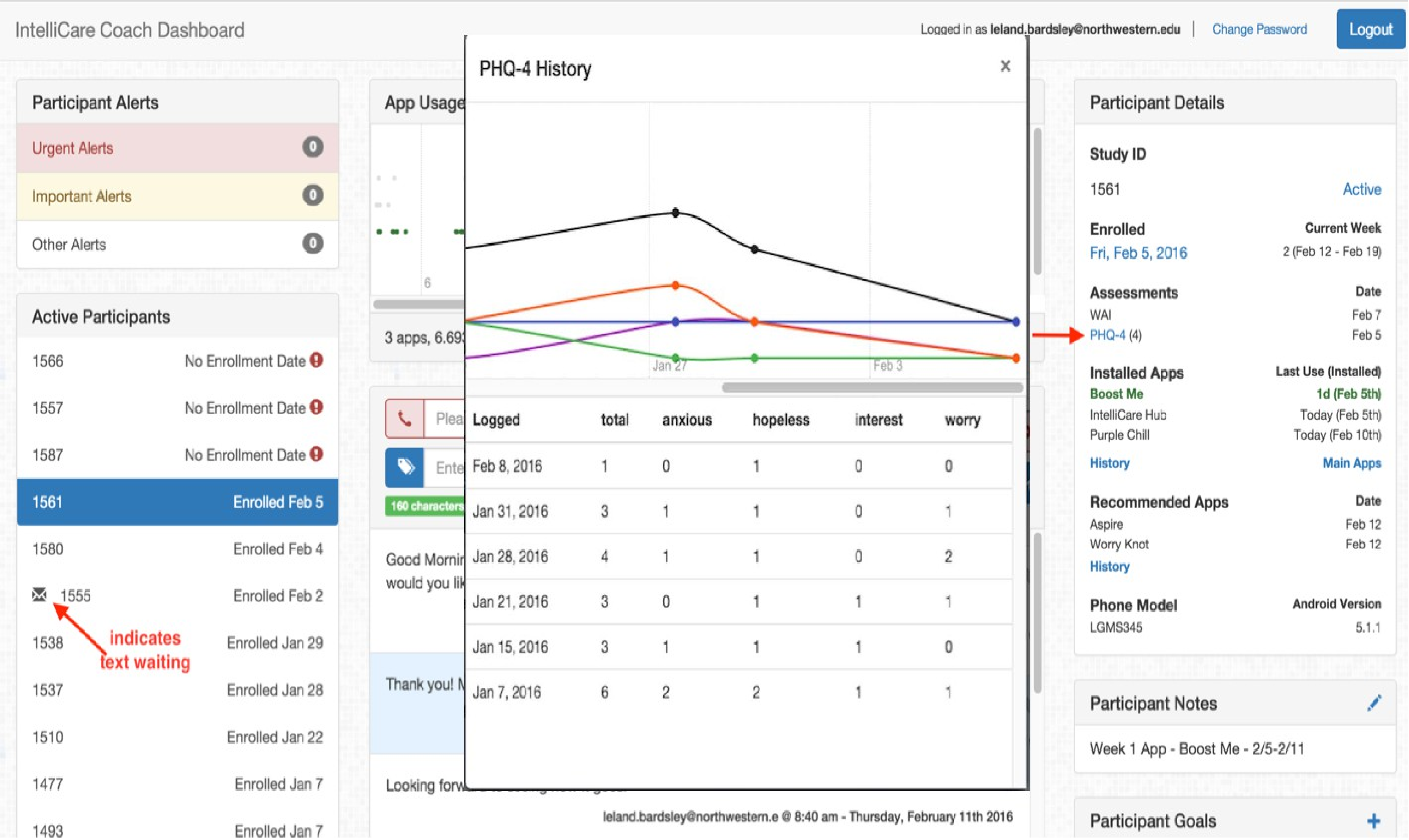

Supplement: Multimedia Appendix 1 [file jmir_v19i1e10_app1.pdf]
